# A Study on Traceable Oxygen-Releasing Microspheres in Combination with Bone Marrow Mesenchymal Stem Cells to Enhance Skin Wound Healing

**DOI:** 10.3390/ijms27114916

**Published:** 2026-05-29

**Authors:** Qianqian Wang, Xiangjie Li, Qing Xu, Yuan Xie, Wenyan Duan, Zhichao Ma, Xue Chen

**Affiliations:** Wuxi School of Medicine, Jiangnan University, Wuxi 214122, China; qqianw163@163.com (Q.W.); lxj98666@163.com (X.L.); 6232817002@stu.jiangnan.edu.cn (Q.X.); 6232811002@stu.jiangnan.edu.cn (Y.X.); 6242811001@stu.jiangnan.edu.cn (W.D.); 6242817003@stu.jiangnan.edu.cn (Z.M.)

**Keywords:** oxygen release, microspheres, fluorescent signals, bone marrow mesenchymal stem cells, wound healing

## Abstract

The treatment of full-thickness skin defects remains a major challenge in clinical medicine. Accelerating wound healing and promoting the restoration of tissue function are of paramount importance. Stem cell therapy has been applied in clinical practice to facilitate wound repair. However, the low survival rate of transplanted stem cells in an ischemic and hypoxic microenvironment severely limits the effectiveness of their clinical application. Microspheres, owing to their excellent biocompatibility and drug delivery capabilities, can serve as effective carriers for oxygen transport. It is worthwhile to evaluate the timing and process of oxygen release under hypoxic conditions. In this study, core–shell structured oxygen-releasing microspheres were prepared and incorporated with the photosensitizer hypericin (HYP) to enable dynamic tracking of the oxygen release process via fluorescent signals. The effects of the oxygen-releasing microspheres on cells under hypoxic conditions were analyzed, focusing primarily on the characterization of the microspheres, their biocompatibility, luminescent properties, and oxygen-releasing capacity. Furthermore, the efficacy of the oxygen-releasing microspheres in combination with bone marrow mesenchymal stem cells (BMSCs) in promoting wound healing was evaluated in vivo. The results indicate that the addition of the microspheres improved cell survival rates in hypoxic environments; meanwhile, their luminescent properties demonstrated the potential of fluorescence intensity as a visual indicator of oxygen release.

## 1. Introduction

The intricate biological process known as wound healing progresses through several distinct phases and cell types, and blood plays a crucial role in supplying oxygen to promote wound healing [1]. Insufficient local blood supply restricts oxygen delivery, thereby inhibiting the migration of inflammatory cells and the proliferation of endothelial cells [2,3,4]. Consequently, ensuring an adequate oxygen supply is crucial during the wound healing process. However, the formation of blood vessels takes time, and during this period, the damaged tissue may undergo necrosis [3,4].

Stem cells possess excellent homing ability; they can migrate to the site of injury and differentiate into all sorts of functional cells required for wound repair, such as keratinocytes, fibroblasts, and vascular endothelial cells, thereby directly participating in the regeneration of skin tissue [5,6]. However, the local ischemic and hypoxic environment at the wound site limits their survival. Research has demonstrated that when injecting mesenchymal stem cells into mice, their survival rate was less than 2% after two weeks [7].

Oxygenation therapy is an effective strategy for treating cellular hypoxia, preventing tissue necrosis, and thereby promoting wound healing [8,9,10,11]. Various forms of oxygenation therapy have been developed. Traditional therapies, such as hyperbaric oxygen therapy, have been used to treat various types of open wounds, including diabetic ulcers [4]. However, hyperbaric oxygen therapy may not be able to sustain oxygen supply to cells under hypoxic conditions. Meanwhile, biomaterials with oxygen-releasing capabilities have been developed to provide oxygen, ensuring cell survival and differentiation to achieve therapeutic effects while preventing tissue damage and cell death caused by hypoxia [12]. Oxygen from organic oxygen-releasing materials, such as hemoglobin and hemoglobin mimetics, can be utilized for wound healing; however, the rate of oxygen release from these materials is too rapid [13]. Inorganic peroxides serve as effective oxygen regulators, facilitating enhanced cellular proliferation, viability, and regenerative processes through controlled oxygen release rate. During aqueous decomposition, select solid inorganic peroxides—calcium peroxide being representative—generate both reactive oxygen species and calcium cations. These decomposition products pose a potential hazard to cells [14].

Blood delivers nutrients and oxygen to tissue cells. When the body sustains an injury, an oxygen-releasing system can supply oxygen to local tissues in place of blood. However, angiogenesis takes 14 days [15,16]. Therefore, when implanting oxygenation systems and cells into wound tissue, it is necessary to investigate whether the duration of oxygen supply is sufficient to sustain the survival of tissue cells in the ischemic region. Monitoring this oxygenation process is of paramount importance. Current methods of oxygen measurement, such as optical oxygen sensors and polarographic needle electrodes, whilst capable of detecting oxygen concentration at specific measurement points, are unable to track the oxygenation process in a specific manner. In contrast to point-measurement techniques, oxygen imaging technologies (such as positron emission tomography (PET), magnetic resonance imaging (MRI), and electron paramagnetic resonance imaging (EPRI)) enable real-time, non-invasive visual monitoring of tissue oxygenation status [10,17]. However, whilst these techniques can detect oxygen within tissues, they struggle to effectively distinguish between exogenously released oxygen and oxygen inherent to the local microenvironment. Consequently, it is necessary to monitor oxygen released specifically from biomaterials in order to assess its relationship with wound healing.

In this study, microspheres capable of spontaneously releasing molecular oxygen were prepared. These microspheres possess photoluminescent properties, enabling researchers to visually monitor the oxygen release process through changes in fluorescence intensity, thereby facilitating the assessment of the relationship between the duration of oxygen release and the survival rate of transplanted stem cells, as well as wound healing outcomes. In vitro experimental results indicate that changes in the microspheres’ fluorescence intensity are highly correlated with their oxygen release behavior. Furthermore, the microspheres demonstrate good biocompatibility. Within oxygen-deficient environments, the oxygen released by the microspheres significantly enhanced the survival rate of BMSCs. To further validate these findings, this study developed a hydrogel composite comprising luminescent oxygen-releasing microspheres and BMSCs, which was then implanted into a mouse model. In vivo results once again confirmed that the oxygen released by the microspheres effectively promoted stem cell survival and migration. In summary, the luminescent oxygen-releasing microspheres developed in this study demonstrate significant potential for promoting rapid wound healing. They not only provide a promising strategy for overcoming the survival challenges faced by stem cell transplantation in ischemic wounds but also enable precise regulation of the microspheres’ oxygen-releasing behavior, thereby offering important technical support for fields such as tissue engineering scaffold design, targeted drug delivery systems, and regenerative medicine therapies.

## 2. Results and Discussion

### 2.1. Fabrication and Characterization of Oxygen-Release Microspheres

The polymer used for the microsphere shell was fabricated by a polymerization reaction, using N-isopropyl acrylamide (NIPAAm), acrylate-oligolactide (AOLA), 2-hydroxyethyl methacrylate (HEMA), and N-acryloxysuccinimide (NAS) as monomers, with benzoyl peroxide (BPO) as the initiator. The polymers (NIPAAm-co-AOLA-co-HEMA-co-NAS) are abbreviated as PNAHN [10]. Previous studies indicate that hydrogel systems synthesized from organic monomers such as NIPAAm, AOLA, and HEMA possess excellent biocompatibility, providing a suitable microenvironment for the proliferation of BMSCs within the hydrogel matrix [10]. Furthermore, the incorporation of NAS endows the polymer side chains with reactive succinimide ester groups, conferring biocatalytic capability and enabling the covalent immobilization of catalase on the microsphere surface. This design facilitates the efficient catalytic conversion of hydrogen peroxide (H_2_O_2_) into O_2_.

**Figure 1:**
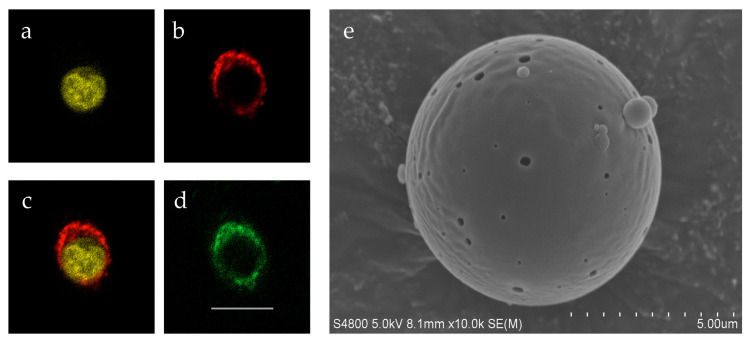
Characterizations of the oxygen-release microspheres. (**a**) The yellow fluorescent image is hypericin (HYP) in the core; (**b**) The red fluorescence is the rhodamine added to the shell; (**c**) Core–shell structure of microspheres; (**d**) Fluorescent image of the oxygen-release microsphere after conjugation with FITC-labelled catalase. Scale bar = 8 μm; (**e**) SEM images of the oxygen-release microsphere.

To achieve the sustained release and visual monitoring of H_2_O_2_, this study formed a complex by chelating it with Polyvinylpyrrolidone (PVP) and the photosensitizer hypericin (HYP). HYP, a naturally derived photosensitizer, is celebrated for its heightened photosensitivity coupled with minimal toxicity, and it has seen clinical use in treating ailments like depression and viral infections [18,19]. This strategy is based on the fact that both H_2_O_2_ and HYP molecules can bind to PVP via hydrogen bonding. With this PVP/H_2_O_2_/HYP composite as the core (Figure 1a) and PNAHN polymer as the shell (Figure 1b), Rhodamine B was incorporated into the shell solution to clearly characterize the core–shell morphology of the microspheres (Figure 1c). To verify the successful immobilization of catalase on the microsphere shell, a coupling reaction was performed using FITC-labeled catalase. Confocal microscope imaging revealed that the FITC-labeled catalase was uniformly distributed across the surface of the microsphere shell (Figure 1d), confirming the effective immobilization of the enzyme molecules. Core–shell microspheres were prepared using the double emulsion method, and their surface morphology was examined using scanning electron microscopy (SEM) (Figure 1e). Microspheres with particle sizes in the range of 5–15 μm were collected by sieving. Microspheres were randomly selected from different scanning electron microscope (SEM) fields of view, and their particle size distribution was statistically analyzed using ImageJ (v1.8.0) software. The results showed that the average diameter of the sieved microspheres was 10.2 ± 2.8 μm.

### 2.2. Cytotoxicity of Oxygen-Release Microspheres

As the predominant cell type in the skin’s connective tissue, fibroblasts play a crucial role in the skin repair process [20,21,22,23]. Given that the microspheres prepared must come into direct contact with wound tissue, and although the literature indicates that hydrogels synthesized from PNAHN polymers exhibit good biocompatibility [2,10], it is nevertheless necessary to conduct a biosafety assessment of the hydrogel microspheres using an in vitro cell culture model prior to animal testing and to preliminarily investigate their effect on accelerating wound healing.

**Figure 2:**
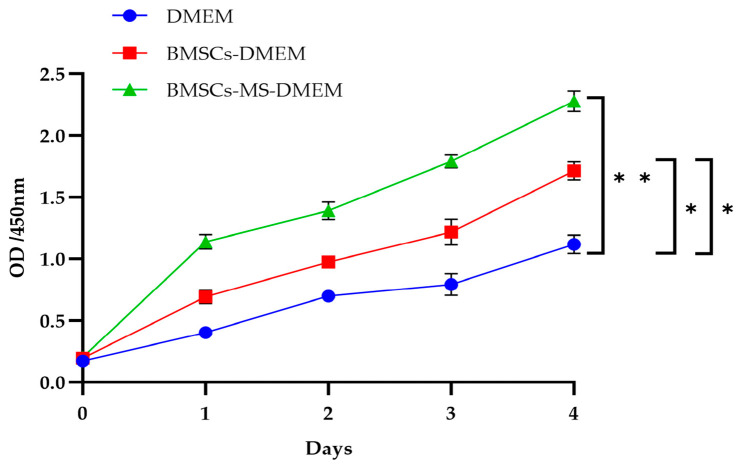
Biocompatibility of microspheres: After co-culturing with L929 cells and BMSCs, cell proliferation in the culture medium with added microspheres was determined by CCK-8 assay.* *p* < 0.05, ** *p* < 0.01.

BMSCs play an important physiological role in tissue angiogenesis and wound healing due to their exceptional multipotent differentiation potential [24,25]. In this study, L929 fibroblasts were used as an in vitro model, and three experimental systems were established: the control group used basic DMEM medium, experimental group 1 added BMSCs to DMEM medium, and experimental group 2 added both BMSCs and microspheres to DMEM medium. Cell Counting Kit-8 (CCK-8) assays conducted over four consecutive days (days 1, 2, 3 and 4) demonstrated that HS and bFGF-HS supported the adhesion and proliferation of L929 and BMSCs. The number of viable cells in all groups increased over time, with the experimental groups showing significantly higher numbers of viable cells than the control group. In contrast, at each observation time point, the number of viable cells in experimental group 2 (containing BMSCs and microspheres) was significantly higher than in the other two groups (Figure 2).

The above results confirm that the prepared microspheres exhibit good cellular compatibility; treatment with either BMSCs or microspheres alone significantly promotes cell proliferation; and the most pronounced enhancement in viable cell numbers is observed when BMSCs are used in combination with microspheres. This phenomenon suggests a potential synergistic mechanism between the microspheres and BMSCs, whereby they jointly facilitate the wound healing process by improving cell proliferation capacity.

Live/dead staining assays were conducted to further evaluate the effects of the microspheres on cells under 1% O_2_ hypoxic conditions (Figure S1). These qualitative images indicate that the majority of BMSCs remained viable when co-cultured with the microspheres under hypoxic conditions. However, due to the lack of quantitative analysis of multiple biological replicates, these images serve only as supplementary qualitative validation.

### 2.3. Monitoring the Fluorescent Signal and Oxygen-Releasing Capacity of the Microspheres

**Figure 3:**
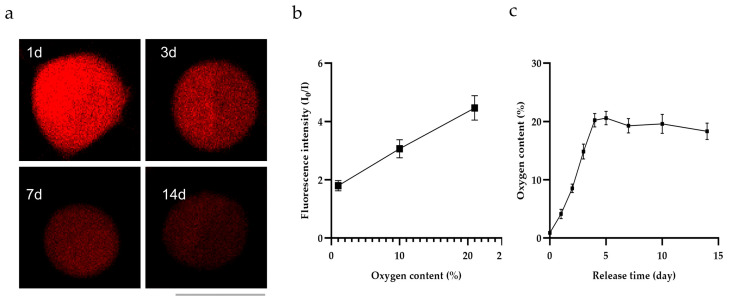
(**a**) In vitro fluorescence signal detection of microspheres: Fluorescence images of microspheres at each time point. Scale bar = 5 μm; (**b**) Calibration curve. I0 is the fluorescence intensity of rhodamine. I is the fluorescence intensity of Ru(ddp); (**c**) Oxygen release kinetics of the microspheres for 2 weeks.

To investigate in depth the mechanism linking the fluorescent signal of the microspheres to oxygen release, this study employed fluorescence imaging techniques to monitor the fluorescence intensity of the microspheres over a 14-day period, with fluorescence images captured on days 1, 3, 7, and 14 (Figure 3a). The results demonstrated that microspheres with longer release periods exhibited weaker fluorescent signals.

To evaluate the oxygen-releasing capacity of the microspheres, we plotted a calibration curve using the oxygen-sensitive luminophore Ru(ddp) and the reference dye rhodamine-B (Figure 3b). We then converted the fluorescence intensities measured at different time points into oxygen concentrations (Figure 3c).

In summary, we have demonstrated that these microspheres can sustain oxygen release for at least 14 days in a hypoxic environment. Fluorescent imaging technology provides a non-invasive method for real-time monitoring of oxygen kinetics in vitro and in vivo, which aids in the design of oxygen-releasing biomaterials for tissue engineering and regenerative medicine.

### 2.4. In Vivo Wound Healing

**Figure 4:**
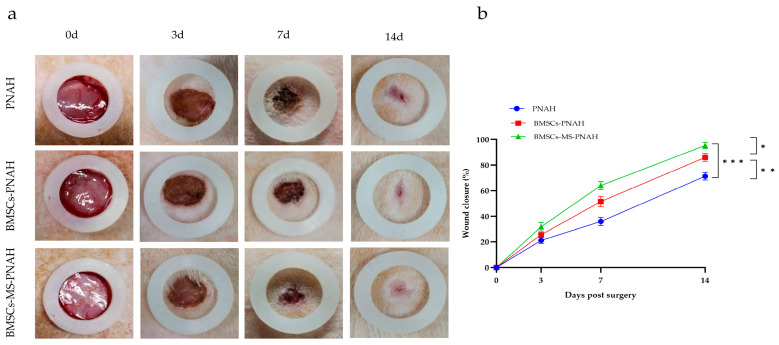
In vivo wound repair process: (**a**). Representative photographs of wounds on days 0, 3, 7, and 14 after injection of PNAH, BMSCs-PNAH, and BMSCs-MS-PNAH hydrogels; (**b**). Wound closure over time in the wound healing model. Data are expressed as mean ± standard deviation (*n* = 3). * *p* < 0.05, ** *p* < 0.01, *** *p* < 0.001.

Due to their excellent biocompatibility, degradability, and low antigenicity, hydrogels have been widely used in research aimed at promoting wound healing [26]. To evaluate the potential role of oxygen-releasing microspheres in promoting wound healing, an animal study was designed for this research (Figure 4a,b). Healthy mice were randomly assigned to three groups: the PNAH hydrogel group (control group), the BMSCs-PNAH hydrogel group (stem cell therapy group), and the BMSCs-MS-PNAH hydrogel group (stem cell-oxygen-releasing microsphere combination therapy group). A circular full-thickness skin defect model with a diameter of 7 mm was created on the back of each mouse. Subsequently, in accordance with the group assignments, the corresponding materials were injected locally into the wound areas. To dynamically monitor the wound regenerative progression, standardized photographic records of the wounds in each group were taken on days 0, 3, 7, and 14 post-surgery. Based on the acquired images, the wound healing rate was calculated (unhealed area/initial area × 100%). ImageJ (v1.8.0) software was used to analyze the images.

The experimental results showed that by day 3 post-treatment, the reduction in wound area was most significant in the BMSCs-MS-PNAH group. On days 7 and 14, the therapeutic effect of the BMSCs-MS-PNAH group was markedly superior to that of the control group. Furthermore, the BMSCs-MS-PNAH group exhibited the fastest wound closure rate and the best healing outcome. The results indicate that BMSCs-MS-PNAH possesses excellent wound-healing properties. Oxygen-releasing microspheres can significantly elevate healing efficiency and restore physiological wound architecture.

### 2.5. Histological Observation

**Figure 5:**
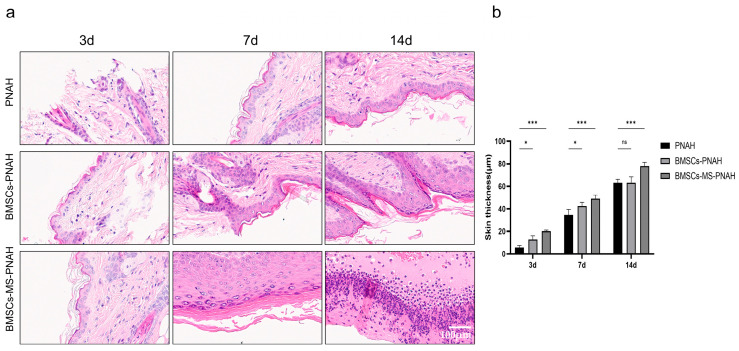
HE staining results: (**a**). HE staining images of wound healing in different treatment groups on the 3rd, 7th and 14th days after treatment. Nuclei are stained blue-purple, while cytoplasm and extracellular matrix are stained pink/red. bar = 100 μm; (**b**). Quantitative statistical analysis of wound healing in different treatment groups by HE staining (*n* = 3). ns, no significant difference; * *p* < 0.05, *** *p* < 0.001.

To systematically evaluate the histological processes of wound healing under different treatment strategies, this study employed hematoxylin and eosin (HE) staining to analyze the microstructure of mouse wound tissue on days 3, 7, and 14 post-surgery (Figure 5a,b). Histological observations revealed that on day 3 post-surgery, the dermis in the PNAH group exhibited structural disorganization accompanied by loose cellular arrangement; in contrast, the dermal tissue structure in the BMSCs-PNAH and BMSCs-MS-PNAH groups was more compact, with significantly improved cell–matrix integration. On day 7 post-surgery, the PNAH group still exhibited delayed dermal structural reconstruction, with extensive empty spaces and immature granulation tissue present beneath the epidermis; the BMSCs-PNAH group showed thickening and extension of the newly formed epidermis, while the BMSCs-MS-PNAH group demonstrated active extracellular matrix regeneration and signs of complete epidermal closure. Day 14 post-surgery: Wounds in all groups were covered by newly formed epidermis, but incomplete epidermal closure persisted in the PNAH group. The BMSCs-MS-PNAH group exhibited the optimal tissue repair status, with collagen deposition in the dermis significantly higher than that in the BMSCs-PNAH group; furthermore, collagen fibers were highly ordered, and the reconstruction of skin appendage structures approached normal histological characteristics.

**Figure 6:**
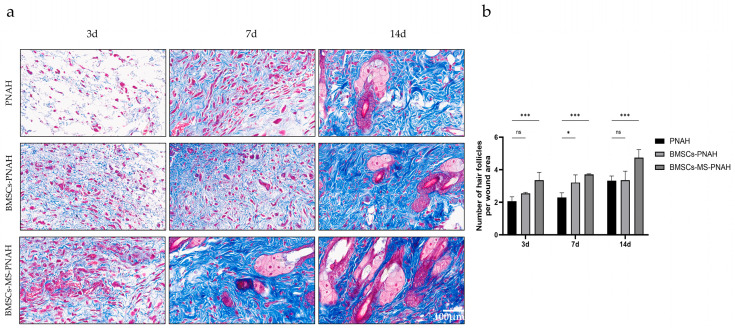
Masson staining results graphs: (**a**). Masson staining images on days 3, 7, and 14 of wound healing in different treatment groups. Collagen fibers are stained blue, while cytoplasm, muscle fibers, and cellular components are stained red. bar = 100 μm; (**b**) Statistical analysis graphs of the number of hair follicles stained by Masson in different treatment groups (*n* = 3). ns, no significant difference; * *p* < 0.05, *** *p* < 0.001.

Masson’s staining is a classic method for assessing the number of collagen fibers and hair follicles. Our comparative analysis revealed that collagen deposition on the wound surface was minimal in all mice on day 3; however, collagen deposition was relatively increased in the BMSCs-MS-PHAN group (Figure 6a,b). By day 7, collagen fibers in the BMSCs-PHAN and BMSCs-MS-PHAN groups appeared more densely packed, and smaller regenerated hair follicles were observed in the BMSCs-MS-PHAN group. Statistical analysis of the Masson staining revealed that the number of hair follicles in the BMSCs-MS-PHAN group was significantly higher than in the PNAH and BMSCs-PHAN groups. By day 14, collagen deposition had further increased in all groups; however, based on observations of collagen distribution and morphology, the collagen fibers in the BMSCs-MS-PHAN group were observed to be intertwined in a reticular structure, with deeper staining, a greater number of regenerated hair follicles, and multiple sebaceous glands in the vicinity. The morphology of the sebaceous gland cells appeared intact, with lighter staining, indicating that they possessed sebum synthesis and secretion functions, and the healing rate was higher. Statistical analysis results showed that the differences were significant.

### 2.6. Oxygen-Release Microspheres Promote Tissue Cell Proliferation

CD29, a member of the integrin family, primarily acts as a receptor for extracellular matrix (ECM) components such as fibronectin and collagen [27,28]. It is extensively involved in mediating cell–cell and cell–matrix interactions and plays a key regulatory role in a variety of important biological processes. CD29 is expressed in a wide range of cell types, including fibroblasts and endothelial cells [29].

**Figure 7:**
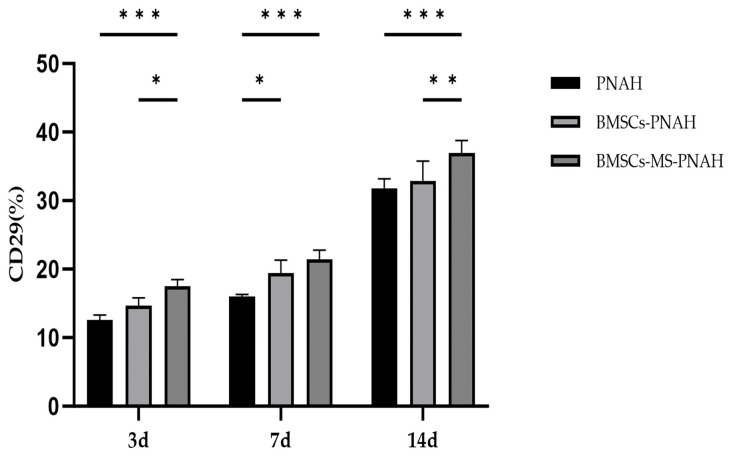
Flow cytometric surface identification: Quantitative analysis of CD29 expression. * *p* < 0.05, ** *p* < 0.01, *** *p* < 0.001.

In this study, flow cytometry was employed to label and detect CD29 expression levels using specific antibodies (Figure 7). The experimental results showed that, compared with the PNAH group and the BMSCs-PNAH group, the BMSCs-MS-PNAH group exhibited a significantly higher percentage of CD29-positive cells at all three time points. This indicates that oxygen-releasing microspheres promote the homing of BMSCs to the wound site and may, through paracrine effects, further activate the expression of fibroblasts and vascular endothelial cells, thereby generating a synergistic repair effect.

These findings suggest that the oxygen released by the microspheres significantly enhances the survival rate of BMSCs, whilst also exerting a positive effect on the proliferation of cells involved in wound repair.

## 3. Materials and Methods

NIPAAm was purified by triple recrystallization from hexane prior to use. HEMA was filtered through a chromatography column containing an inhibitor removal agent [30]. Bovine liver catalase (3500 units/mg), HYP (Mackin, Shanghai, China), and the CCK-8 (Beyotime, Shanghai, China) were used directly without further purification. The other materials used in this study were purchased from Aladdin (Shanghai, China).

### 3.1. Fabrication of Oxygen-Release Microspheres

The microspheres had a core–shell structure [2]. The shell of the microspheres was synthesized by free radical polymerization of NIPAAm, AOLA, HEME, and NAS in a ratio of 50:25:5:20 as previously reported [31,32,33,34,35,36,37].

The core is a stable PVP/H_2_O_2_ complex. Mix H_2_O_2_ with PVP in a 4.5:1 ratio and stir vigorously overnight at 4 °C. Subsequently, add HYP to the PVP/H_2_O_2_ solution according to the ratio of 10 mg HYP per gram of PVP. Stir the mixture overnight in a refrigerator at 4 °C. Dialyze in deionized water for three days to eliminate free HYP.

Microspheres were prepared using a double emulsion method [38]. Briefly, a 5% organic phase was prepared by dissolving PNAHN in dichloromethane. Subsequently, the aqueous component (PVP/H_2_O_2_/HYP) was rapidly injected into the organic phase and processed ultrasonically with an ultrasonic liquid processor (Scientz, Ningbo, China). The oil-in-water primary emulsion was injected into a polyvinyl alcohol solution to form a water-in-oil-in-water double emulsion. After stirring for 3 h, dichloromethane was removed, and microspheres were collected by centrifugation. Morphology and size were characterized using SEM (S-4800, Hitachi, Tokyo, Japan). Rhodamine B fluorescence marker was introduced to the PNAHN/dichloromethane solution to validate the core–shell configuration. Fluorescence images were acquired via confocal microscopy (Leica TCS SP5, Leica, Mannheim, Germany). Catalase was immobilized on the microsphere surface via reaction with NAS. To verify catalase conjugation, the enzyme was labeled with fluorescein isothiocyanate (FITC). Fluorescence labeling was observed using confocal microscopy.

### 3.2. Preparation of Injectable Hydrogels

Polymerization of NIPAAm, AOLA, and HEME at 86:4:10 mol% under radical conditions yielded the crosslinked hydrogel structure (abbreviated as PNAH). The hydrogel is employed to deliver microspheres and BMSCs to hypoxic tissues.

### 3.3. CCK-8 Assay

Cell proliferative capacity was quantified via the CCK-8 assay in co-cultures of microspheres with L929 cells and BMSCs. First, cells (5 × 10^4^) were seeded into 96 wells and allowed to adhere for 12 h. Subsequently, the original culture medium in the experimental group was replaced with fresh medium containing BMSCs (2 × 10^3^) cell suspension supplemented with or without oxygen-releasing microspheres (40 mg/mL), and the plates were incubated at 37 °C. CCK-8 solution was added to each well at 24, 48, 72, and 96 h, followed by incubation in the dark for 2 h. Absorbance values were measured at 450 nm using a microplate reader (Epoch, BioTek, VT, USA).

### 3.4. Oxygen Release Capacity of Microspheres and Fluorescence Assay

Polydimethylsiloxane (PDMS) membranes embedded with the oxygen-sensitive luminescent agent Ru(ddp) and the oxygen-insensitive fluorescent agent Rhodamine B were placed in a 96-well plate, and 200 μL of DPBS containing microspheres in suspension was added to each well. The fluorescence intensities of Ru(ddp) and Rhodamine B were measured at different time points. Subsequently, the fluorescence intensities were converted to oxygen concentrations by measuring standard curves obtained under 1%, 10%, and 21% oxygen conditions [10,33].

Under 1% O_2_ concentration, oxygen-releasing microspheres (40 mg/mL) were added to the cell culture medium. BMSCs were co-incubated with the oxygen-releasing microspheres. Diluted DCFH-DA was added and incubated for 30 min to allow the probe to enter the cells and undergo oxidation. Observation was conducted under fluorescence microscopy (Axio Imager Z2, Zeiss, Oberkochen, Germany) with an excitation wavelength of 488 nm and an emission wavelength of 530 nm. The DPBS solution containing the microspheres was added to a 96-well plate. The culture dish was placed in a 37 °C incubator with 1% O_2_. At 7 days, the supernatant was collected, and fluorescence images of the microspheres were captured. The images were observed using a confocal microscope.

### 3.5. Wound Healing

Six- to eight-week-old female C57BL/6 mice were purchased from Shanghai SLAC Laboratory. All experimental procedures were approved by the Animal Ethics Committee of Jiangnan University. Animal ethics approval number: JN.No.20240930c0721110[508]. Mice were anesthetized with tribromoethanol, and a 7 mm diameter full-thickness skin defect model was prepared on the dorsal region. Following successful wound creation, 200 μL of PNAH hydrogel (6 wt% DPBS), PNAH hydrogel containing BMSCs (6 wt% DPBS, 2 × 10^6^/mL BMSCs), and PNAH hydrogel containing microspheres and BMSCs (6 wt% DPBS, 2 × 10^6^/mL BMSCs, and 50 mg/mL microspheres) were locally injected. Wound photographs were taken on days 3, 7, and 14 and analyzed using ImageJ (v1.8.0) software.

### 3.6. Histological Staining

Tissue was harvested from the dorsal region and promptly fixed in 4% paraformaldehyde solution, followed by paraffin embedding. Sections were dewaxed using xylene and washed with graded ethanol solutions. Hematoxylin and eosin staining was performed for 5–10 min, after which morphological evaluation was conducted. Specimens were first stained with Alcian blue-acid fuchsin for 5–10 min, followed by counterstaining with aniline blue. Using Masson’s trichrome staining, hair follicle regeneration was observed at the wound site. Collagen deposition was also evident in this area. Observation and imaging of stained areas were performed using a slide scanner (Panoramic MIDI, 3DHISTECH Ltd., Budapest, Hungary).

### 3.7. Flow Cytometry

Tissue samples were taken from mouse wounds at 3, 7, and 14 days post-injury; these were enzymatically digested, sieved, centrifuged, and washed, and the cell concentration was adjusted to 1 × 10^7^ cells/mL. The cells were resuspended in 100 µL of diluted primary antibody solution (CD21, 1:1000). Subsequently, the cells were resuspended in 100 µL of diluted fluorescently labeled secondary antibody and incubated at 4 °C in the dark. The cells were resuspended in PBS, and a flow cytometer was used to analyze the cell surface markers. The data obtained were analyzed and processed.

### 3.8. Statistical Analysis

All data are expressed as mean ± standard deviation (SD). Statistical significance was determined using two-way analysis of variance (ANOVA) using GraphPad Prism Software (V. 10). Differences were considered significant at *p* < 0.05, *p* < 0.01, and *p* < 0.001 and are indicated by *, **, and ***, respectively.

## 4. Conclusions

Photoluminescent oxygen-releasing microspheres, combined with BMSCs, markedly improve cell survival under hypoxia and accelerate wound healing in mice. The microspheres’ photoluminescent properties allow non-invasive monitoring of oxygen release, offering a valuable tool for optimizing oxygen delivery in tissue reconstruction procedures and regenerative biomedicine.

## Data Availability

The original contributions presented in this study are included in the article/Supplementary Materials. Further inquiries can be directed to the corresponding author.

## Supplementary Materials

The following supporting information can be downloaded at: https://www.mdpi.com/article/10.3390/ijms27114916/s1.
