# Supplementary material for: A Study on Traceable Oxygen-Releasing Microspheres in Combination with Bone Marrow Mesenchymal Stem Cells to Enhance Skin Wound Healing
Source: Int J Mol Sci. 2026 May 29;27(11):4916. doi: 10.3390/ijms27114916 (PMC13256253; doi:10.3390/ijms27114916)

Figure S1. Fluorescence images of live and dead cells: co-culture of microspheres with BMSCs under hypoxic conditions, day 7, fluorescence images after reaction of Calcein-AM and PI with the cells, green labelled live cells, red labelled dead cells. bar=20um

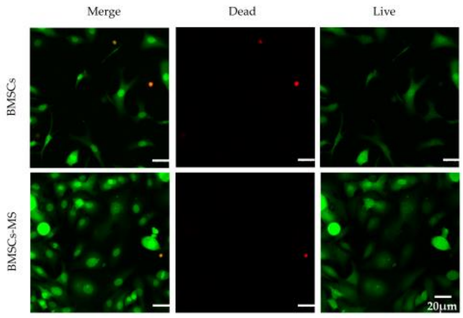

Supplement: Supplementary file 1 [file ijms-27-04916-s001.zip › ijms-4283214-supplementary.pdf]
